# Predictors of Mortality in Hospitalized COVID-19 Patients Complicated With Hypotension and Hypoxemia: A Retrospective Cohort Study

**DOI:** 10.3389/fmed.2021.753035

**Published:** 2021-10-28

**Authors:** Baoni Sun, Hai Wang, Junhua Lv, Honghong Pei, Zhenghai Bai

**Affiliations:** ^1^Emergency Department, The Second Affiliated Hospital of Xi'an Jiaotong University, Xi'an, China; ^2^Department of Hepatobiliary Surgery, The First Affiliated Hospital of Xi'an Jiaotong University, Xi'an, China

**Keywords:** COVID-19, hypotension, hypoxemia, prognosis, predictors

## Abstract

**Introduction:** COVID-19 patients with hypotension and hypoxemia had a significantly worse outcome. The purpose of this research was to ascertain the risk factors affecting the prognoses of these patients and to develop appropriate prognostic prediction tools.

**Methods:** From March 1, 2020, to April 16, 2020, a retrospective cohort analysis of COVID-19 patients with hypotension and hypoxemia was performed. The univariate and multivariate analyses were performed to identify the associated risk factors influencing the prognosis of COVID-19 patients with hypotension and hypoxemia, and the selected variables were then utilized to construct and validate the prediction model for these patients.

**Results:** Three hundred and twenty-seven COVID-19 patients with hypotension and hypoxemia who met the inclusion and exclusion criteria were included in this study. Age, temperature, troponin, and blood glucose were related to mortality in COVID-19 patients with hypotension and hypoxemia in both univariate and multivariate analyses. The MFP model (multiple fractional polynomial model), full model, and stepwise model were utilized to build the prediction model, and their AUCs were, respectively, 0.902 (0.868, 0.936), 0.902 (0.868, 0.936), and 0.902 (0.868, 0.936). Because the sample size for this research was limited, we utilized bootstrapping for internal validation. The AUCs of Bootstrap full and Bootstrap stepwise were 0.902 (0.867, 0.936) and 0.902 (0.868, 0.936), respectively.

**Conclusion:** Age, temperature, troponin, and blood glucose levels were associated with mortality in COVID-19 patients with hypotension and hypoxemia. Additionally, the prediction model developed using the variables above showed a high predictive value for predicting the prognosis of these individuals.

## Introduction

COVID-19 has caused about 170 million illnesses and millions of deaths since its breakout in 2019. What is more concerning is that, although the COVID-19 vaccine has been used in clinics, the number of newly diagnosed cases continues to grow by the hundreds of thousands each day. Approximately 80% of individuals infected with COVID-19 were mild cases, whereas approximately 14% acquired severe cases (1, 2), and 5% of cases progressed to severe cases (2). Severe patients had a mortality rate of above 50%, which was much higher than the rate for other patients (3). Hypotension is a common symptom of COVID-19, with a prevalence of 30–40% (1, 3, 4). It is also the most prevalent complication among dying patients (3). COVID-19 is an acute respiratory infectious illness that often infects the lungs. Around 36% of these individuals may have hypoxemia, which may progress to acute respiratory distress syndrome (ARDS) (5). Hypotension or hypoxemia are both independently associated with COVID-19 mortality (6–9). Hypotension or hypoxemia may both substantially aggravate the status of COVID-19 patients. Some studies had demonstrated that patients who simultaneously suffered from hypoxia and hypotension were with a poor prognosis. For example, a study about brain injury found that patients with hypoxia and hypotension had a worse prognosis (10), while another study about premature newborns found that patients with hypoxia and hypotension had a 53% increased risk of death, compared to those with hypoxia or hypotension alone (11). At the same time, we also found that patients who simultaneously suffered from hypoxia and hypotension had the highest mortality. For more details, please see Supplementary Table 1.

However, studies on COVID-19 individuals who complicated with hypotension and hypoxemia and a method for predicting their prognosis are missing. As a result, it is essential to investigate the risk factors associated with poor prognosis in COVID-19 patients with hypotension and hypoxemia and develop an efficient method for predicting these patients' prognoses.

## Materials and Methods

### Study Design

A retrospective cohort study.

### Objective

To investigate the risk factors influencing the prognosis of COVID-19 patients with hypotension and hypoxemia and to develop an effective prediction tool.

### Data Source

The data in this study were provided by Altschul, David, and stored in Dryad Database (https://datadryad.org/stash/dataset/doi:10.5061/dryad.7d7wm37sz) (12, 13).

### The Definition of Hypotension

Mean arterial pressure (MAP) <65 mmHg was considered hypotension.

### The Definition of Hypoxemia

Hypoxemia was characterized as a blood oxygen saturation (SPO2) of <90%.

### Inclusion Criteria

(1) Patients were diagnosed with COVID-19 by RT-PCR and admitted to hospital for treatment; (2) patients were older than 18 years old; (3) patients complicated with hypotension and hypoxemia; (4) For patients admitted to hospital many times, only the last admission was included for analysis.

### Exclusion Criteria

The value of MAP or blood oxygen saturation was not available.

### Participants

From March 1, 2020, to April 16, 2020, Patents infected with COVID-19 diagnosed by RT-PCR were collected. The follow-up period concluded on May 7, 2020. A total of 4,711 cases verified by COVID-19 and 327 cases complicated with hypotension and hypoxemia met the inclusion and exclusion criteria and were included in this research.

### Ethics Statement

New ethics approval was not applicable since the original author had already obtained ethical approval when conducting this study. Permission to participate was also not appropriate since our analysis was a retrospective examination of data reuse, and the patients' messages were anonymous.

### Clinical and Biochemical Data Collection

On admission, demographic, and clinical data such as age, race, temperature, MAP, SPO2, and comorbidities (myocardial infarction, peripheral vascular disease, congestive heart failure, cerebrovascular disease, dementia, COPD, and diabetes) were collected. The following biochemical data were also collected on admission, including white blood cells (WBC), troponin, ferritin, creatinine, procalcitonin, c-reactive protein, IL6, ALT, AST, glucose, BUN, INR, platelets, and D-Dimer. Death-related data were gathered through hospital death registration and death registration in the national death registry.

### Statistical Analysis

For measurement data, the median (Q1–Q3) was utilized, whereas for counting data, the *n* (percent) was used. Univariate analysis and multivariate Cox regression analysis were conducted to identify potential risk factors associated with the mortality of COVID-19 patients complicated with hypotension and hypoxemia. The receiver operating characteristic (ROC) curve analysis was used to further assess the predictive usefulness of the risk factors on the mortality of COVID-19 patients who had hypotension and hypoxemia. Additionally, the variables selected by univariate analysis and multi-factor analysis multivariate Cox regression analysis were utilized to construct appropriate prediction models using the multiple fractional polynomial (MFP) model, full model, and the stepwise model, with internal verification performed via bootstrapping. All statistical analysis was carried out by EmpowerStats 2.0 (Copyright 2009 X&Y Solutions, Inc.) and R software. *P* < 0.05 was statistically significant.

## Results

### The Clinical Characteristics of Patients

A total of 327 patients complicated with hypotension and hypoxemia met the inclusion and exclusion criteria were included in this study. The median age was 70.5 years (IQR, 62–80 years), the median temperature was 37.17°C (IQR, 36.72–38.00°C), the median MAP was 45.84 mmHg (IQR, 29.25–55.75 mmHg) and the median SPO2 was 79.00% (IQR, 60.75–85.00%). The race including Black (*n* = 115), White (*n* = 39), Asian (*n* = 7), Latino (*n* = 127), and Other (*n* = 39). Comorbidities among these patients included myocardial infarction (*n* = 14, 4.28%), peripheral vascular disease (*n* = 66, 20.18%), congestive heart failure (*n* = 42, 12.84%), cerebrovascular disease (*n* = 35, 10.70%), dementia (*n* = 23, 7.03%), COPD (*n* = 19, 5.81%), and diabetes (*n* = 56, 17.13%) (see Table 1).

**Table 1:** The clinical characteristics of patients.

**Variables**	**Median (Q1–Q3)/*N* (%)**
Age, year	70.50 (62.00–80.00)
Temperature, °C	37.17 (36.72–38.00)
Mean arterial pressure, mmHg	45.84 (29.25–55.75)
SPO_2_, %	79.00 (60.75–85.00)
**Race**	
Black, *n* (%)	115 (35.17%)
White, *n* (%)	39 (11.93%)
Asian, *n* (%)	7 (2.14%)
Latino, *n* (%)	127 (38.84%)
Other, *n* (%)	39 (11.92%)
Myocardial infarction, *n* (%)	14 (4.28%)
Peripheral vascular disease, *n* (%)	66 (20.18%)
Congestive heart failure, *n* (%)	42 (12.84%)
Cerebrovascular disease, *n* (%)	35 (10.70%)
Dementia, *n* (%)	23 (7.03%)
COPD, *n* (%)	19 (5.81%)
Diabetes, *n* (%)	56 (17.13%)
WBC, 10^9^/L	8.90 (6.07–12.83)
Troponin, ng/ml	0.01 (0.01–0.05)
Ferritin, μg/L	713.50 (0.00–1838.50)
Creatinine, μmol/L	1.37 (0.82–2.10)
Procalcitonin, ng/ml	0.20 (0.00–1.10)
C-Reactive protein, mg/L	15.10 (4.15–26.63)
IL6, pg/ml	0.00 (0.00–74.97)
ALT, U/L	28.50 (17.00–47.25)
AST, U/L	54.00 (29.75–86.50)
Glucose, mg/dl	117.00 (0.00–177.00)
BUN, mg/dl	20.00 (0.00–50.25)
INR	1.10 (1.00–1.30)
Platelets, 10^9^/L	204.00 (149.75–276.00)
D-D dimer, mg/L	204.00 (149.75–276.00)
Death, *n* (%)	176 (53.82%)

### The Results of Univariate Analysis and Multivariate Cox Regression Analysis

Age, Temperature, Black, Troponin, Creatinine, WBC, C-Reactive protein, ALT, Glucose, BUN, INR, Platelets, and D-dimer were all related to mortality of COVID-19 individuals with hypotension and hypoxemia, according to univariate analysis. The multivariate Cox regression analysis revealed that only age, temperature, troponin, and blood glucose were related to the mortality of COVID-19 patients with hypotension and hypoxemia. The HR of them were, respectively, 1.027 (1.012, 1.042), 1.041 (1.023, 1.059), 2.951 (1.019, 8.543) and 1.002 (1.001, 1.004) (see Table 2).

**Table 2:** The results of the univariate and multivariate cox regression analysis.

**Exposure**	**Univariate HR (95% CI), *P***	**Multivariate HR (95% CI), *P***
Age, year	1.036 (1.023, 1.048), <0.001	1.027 (1.012, 1.042), <0.001
Temperature, °C	1.050 (1.036, 1.065), <0.001	1.041 (1.023, 1.059), <0.001
**Race**		
Black, *n* (%)	0.696 (0.499, 0.971), 0.033	1.060 (0.625, 1.798), 0.828
White, *n* (%)	1.332 (0.871, 2.036), 0.186	1.337 (0.770, 2.321), 0.303
Asian, *n* (%)	1.205 (0.446, 3.253), 0.713	2.748 (0.843, 8.962), 0.094
Latino, *n* (%)	1.121 (0.830, 1.512), 0.456	1.507 (0.925, 2.458), 0.100
Myocardial infarction, *n* (%)	0.505 (0.223, 1.144), 0.102	0.470 (0.150, 1.479), 0.197
Peripheral vascular disease, *n* (%)	0.669 (0.431, 1.038), 0.073	1.083 (0.624, 1.879), 0.778
Congestive heart failure, *n* (%)	0.883 (0.580, 1.343), 0.560	1.190 (0.634, 2.236), 0.588
Cerebrovascular disease, *n* (%)	0.888 (0.564, 1.398), 0.609	0.812 (0.435, 1.516), 0.513
Dementia, *n* (%)	0.892 (0.515, 1.544), 0.682	0.588 (0.281, 1.233), 0.160
COPD, *n* (%)	0.977 (0.530, 1.801), 0.941	1.017 (0.440, 2.354), 0.968
Diabetes, *n* (%)	0.704 (0.461, 1.077), 0.106	0.809 (0.479, 1.365), 0.427
Procalcitonin, ng/ml	1.003 (0.981, 1.025), 0.794	0.979 (0.952, 1.006), 0.129
Troponin, ng/ml	2.907 (1.376, 6.141), 0.005	2.951 (1.019, 8.543), 0.046
Ferritin, μg/L	1.000 (1.000, 1.000), 0.110	1.000 (1.000, 1.000), 0.828
Creatinine, μmol/L	1.084 (1.041, 1.128), <0.001	1.031 (0.957, 1.112), 0.420
WBC, × 10^9^/L	1.027 (1.015, 1.040), <0.001	0.979 (0.948, 1.012), 0.207
C-Reactive protein, mg/L	1.023 (1.013, 1.032), <0.001	1.008 (0.993, 1.024), 0.289
IL6, pg/ml	1.000 (1.000, 1.000), 0.952	1.000 (1.000, 1.000), 0.503
ALT, U/L	1.005 (1.003, 1.008), <0.001	1.002 (0.998, 1.006), 0.294
Blood glucose, mg/dl	1.003 (1.002, 1.004), <0.001	1.002 (1.001, 1.004), 0.001
BUN, mg/dl	1.013 (1.009, 1.017), <0.001	1.004 (0.998, 1.011), 0.185
INR	1.385 (1.217, 1.576), <0.001	1.081 (0.833, 1.404), 0.556
Platelets, 10^9^/L	1.003 (1.002, 1.004), <0.001	1.000 (0.998, 1.002), 0.997
D-D dimer, mg/L	1.049 (1.030, 1.069), <0.001	1.017 (0.993, 1.041), 0.169

### Predictive Value of Age, Temperature, Blood Glucose, and Troponin by Operating Receiver Curve

The Area under the ROC curve (AUC) of age, temperature, troponin, and blood glucose for predicting the mortality of COVID-19 patients with hypotension and hypoxemia were, respectively, 0.714 (0.658, 0.770), 0.862 (0.821, 0.904), 0.635 (0.578, 0.691), and 0.729 (0.677, 0.782). The total AUC was 0.902 (0.868, 0.936) (see Table 3).

**Table 3:** Predictive value of age, temperature, glucose, and troponin.

**Variable**	**Best threshold**	**Specificity**	**Sensitivity**	**Accuracy**	**AUC (95%CI)**
Age, year	68.5	0.689	0.659	0.673	0.714 (0.658, 0.770)
Temperature, °C	36.2	0.813	0.871	0.844	0.862 (0.821, 0.904)
Glucose, mg/dl	134.5	0.808	0.466	0.624	0.635 (0.578, 0.691)
Troponin, ng/ml	0.005	0.477	0.886	0.697	0.729 (0.677, 0.782)
Total	–	0.840	0.877	0.860	0.902 (0.868, 0.936)

### The Construction and Verification of the Prediction Model

**Figure 1:**
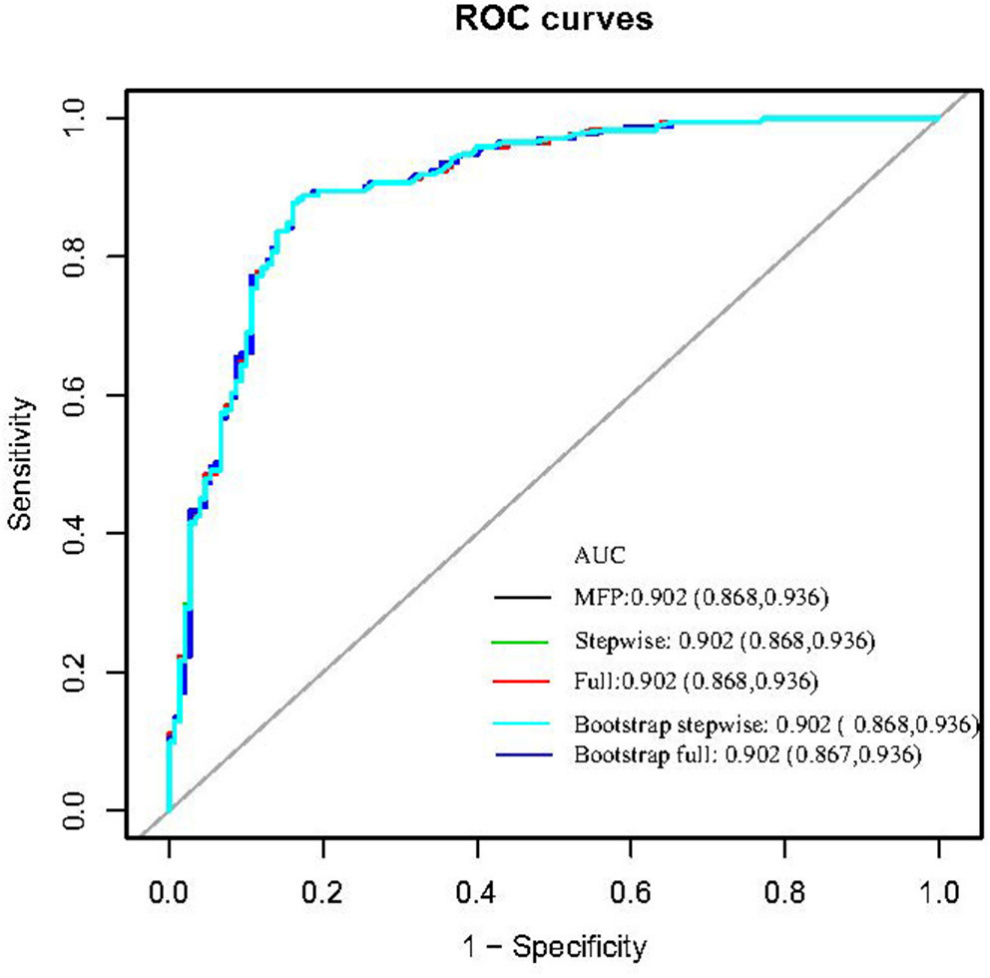
The ROC curve of the predictive model in training cohort and validation cohort.

The four variables (age, temperature, blood glucose, and troponin) selected by univariate analysis and multi-factor analysis were used to construct and verify the prediction model. We built the prediction model in the following three ways: MFP Model, Full Model, and Stepwise Model. The AUC of them were respectfully 0.902 (0.868, 0.936), 0.902 (0.868, 0.936), and 0.902 (0.868, 0.936). Due to the limited sample size of this research, we used adopted bootstrapping for internal verification. The AUC of Bootstrap full and Bootstrap stepwise were, respectively, 0.902 (0.867, 0.936) and 0.902 (0.868, 0.936). We used the stepwise model as our goal model since the stepwise model just had two variables: age and body temperature (see Table 4; Figure 1).

**Table 4:** The results of predictive models.

**Models**	**AUC (95%CI)**	**Specificity**	**Sensitivity**	**Accuracy**
MFP model	0.902 (0.868, 0.936)	0.840	0.877	0.860
Full model	0.902 (0.868, 0.936)	0.840	0.877	0.860
Stepwise model	0.902 (0.868, 0.936)	0.840	0.877	0.860
Bootstrap full	0.902 (0.867, 0.936)	0.840	0.877	0.860
Bootstrap stepwise	0.902 (0.868, 0.936)	0.840	0.877	0.860

*MFP model, multiple fractional polynomial model; stepwise selected model, stepwise model; bootstrap full, full model from bootstrap; bootstrap stepwise, BS stepwise, stepwise most selected model from bootstrap*.

## Discussion

In this study, we found that age, temperature, troponin, and blood glucose were associated with the mortality of COVID-19 patients complicated with hypotension and hypoxemia. The four variables were used to construct the prognosis prediction model for these patients, and we discovered that it had a high predictive value, the AUC was higher than 0.9, and high sensitivity and specificity.

Numerous studies had shown that age was an independent risk factor for a poor outcome with COVID-19 (14–17). The following are possible explanations: chronic illnesses such as hypertension, diabetes, and coronary heart disease become increasingly prevalent as people become older (18); the elderly's baseline level of proinflammatory cytokines in tissues and circulation increases with age, and the body's immune response to pathogenic threats or tissue damage is also delayed (19); the angiotensin-converting enzyme 2 (ACE-2) receptor is required for the COVID-19 virus to enter cells (20). Because the elderly have a greater incidence of hypertension, diabetes, cardiovascular and cerebrovascular disease, they use more ACEI and ARBs, which up-regulate the ACE-2 receptor (21). The increased expression of the ACE-2 receptor promotes the entrance of the COVID-19 virus into older individuals and contributes to their deterioration. According to the findings of this study, the mortality risk of COVID-19 patients with hypotension and hypoxemia rose by 2.7% for every year of age, which was consistent with prior research findings.

One of the most frequent symptoms in COVID-19 patients is fever. A study of 9,417 COVID-19 patients discovered that more than half of the patients had a fever when admitted to the hospital, with the fever rate reaching 78.5% throughout hospitalization (22). At the same time, body temperature correlated with the severity of COVID-19. Jiangshan Lian's study comprised 788 COVID-19 patients and discovered that older patients were more likely to have a high fever, and those with fever had a poorer prognosis (23). Furthermore, Deborah H L Ng's study discovered that 12.7% of COVID-19 patients had a long-term fever, and patients with long-term fever had a greater inflammatory response, as well as a higher risk of hypoxemia and mechanical ventilation than other patients (24).

Myocardial damage is another frequent consequence in COVID-19 patients, accounting for about 30% of COVID-19 inpatients (25). Alessandro Maino performed research on the epidemiology and features of COVID-19 myocardial damage and discovered that myocardial injury was one of the most frequent COVID-19 consequences. Furthermore, the researchers discovered that older patients were more vulnerable to myocardial damage, that patients with myocardial injury had a higher ICU occupancy rate, and that patients with myocardial injury had higher mortality (26). Troponin has been identified as a marker of myocardial damage. According to this research, the greater the troponin, the poorer the prognosis of COVID-19 patients, which was consistent with the previous study. In addition to COVID-19-induced myocardial damage, hypotension, or hypoxemia may cause or exacerbate the myocardial injury, and myocardial injury can also develop hypotension and hypoxemia.

Barrak Alahmad's research comprised 417 COVID-19 participants. The research discovered that the greater the blood glucose, the more severe the COVID-19 patients were. The research also discovered that even a slight rise in blood glucose levels within the normal range was associated with a worsening of the patients' outcomes. In a systematic review and meta-analysis of COVID-19 patients, Juan Chen discovered that the greater the blood glucose, the more severe the illness and the worse the prognosis (27).

To summarize, the four variables: age, temperature, troponin, and blood glucose, were significantly related to the prognosis of COVID-19 patients with hypotension and hypoxemia. The prediction model for forecasting the prognosis of these individuals was reasonable and reliable using the four variables mentioned above.

## Limitations of Research

This study is a retrospective cohort study, so the conclusions of this study need to be further verified in prospective studies; as the sample size of COVID-19 patients complicated with hypotension and hypoxemia was relatively small, only internal verification was used to verify the model, so the model obtained in this study must be confirmed in additional studies; Only the last admission was included for analysis if the patient was admitted multiple times; the conclusions of this study may be overestimated; the population constructed by this research model was COVID-19 patients complicated with hypotension and hypoxemia. Therefore, the model's area of applicability in this study was restricted.

## Conclusion

Age, temperature, troponin, and blood glucose levels were associated with mortality in COVID-19 patients with hypotension and hypoxemia. Additionally, the prediction model developed using the variables above showed a high predictive value for predicting the prognosis of these individuals.

## Data Availability Statement

The datasets presented in this study can be found in online repositories. The names of the repository/repositories and accession number(s) can be found at: https://datadryad.org/stash/dataset/doi:10.5061/dryad.7d7wm37sz.

## Ethics Statement

New ethics approval was not applicable since the original author had already obtained ethical approval when conducting this study. Permission to participate was also not appropriate since our analysis was a retrospective examination of data reuse, and the patients' messages were anonymous. Written informed consent for participation was not required for this study in accordance with the national legislation and the institutional requirements.

## Author Contributions

BS participated in the research design, data analysis, and writing of the paper. HW participated in data analysis and revising of the paper. JL and HP participated in the improving and revising of the paper. ZB provided substantial advice in designing the study and assisting in the division of labor, writing, and revising the paper. All authors contributed to the article and approved the submitted version.

## Funding

Xi'an City Science and Technology + Action Plan—medical research project, Grant/Award Number: 2019115713XY012SF049.

## Conflict of Interest

The authors declare that the research was conducted in the absence of any commercial or financial relationships that could be construed as a potential conflict of interest.

## Publisher's Note

All claims expressed in this article are solely those of the authors and do not necessarily represent those of their affiliated organizations, or those of the publisher, the editors and the reviewers. Any product that may be evaluated in this article, or claim that may be made by its manufacturer, is not guaranteed or endorsed by the publisher.
